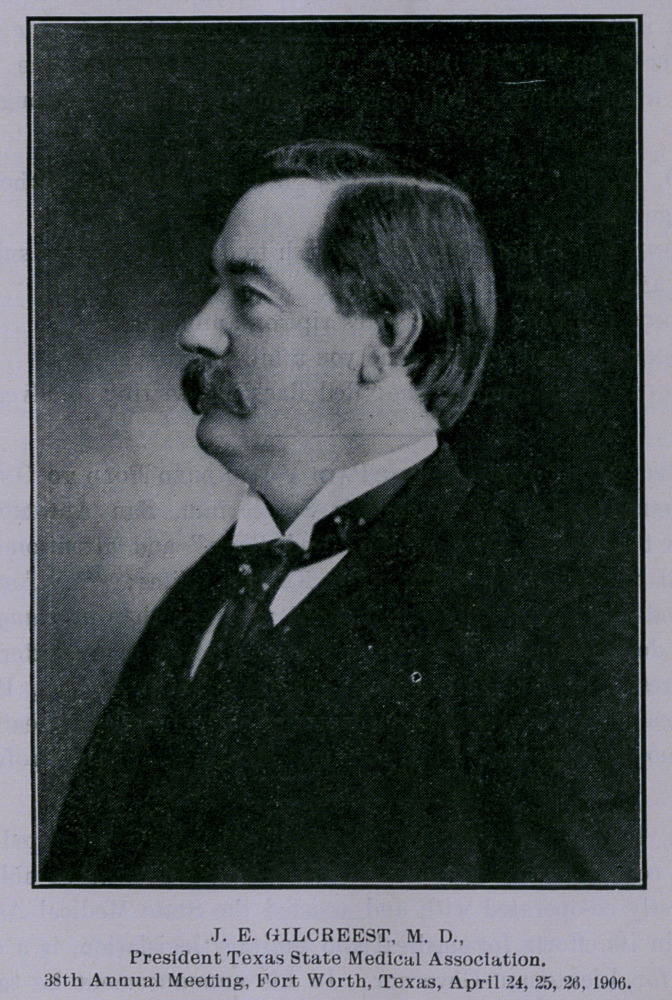# Editorialets

**Published:** 1906-04

**Authors:** 


### Editorialets.

THE PROGRAM for the big Fort Worth meeting appears in full in the State Association's journal, and a copy has been put in the hands of every member. Reprints, as usual, will be distributed at the meeting. Heretofore members have looked to the "Red Back" for the announcement and program; but it has been superceded,

J. E. GILCREEST, M. D.,

President Texas State Medical Association.

38th Annual Meeting, Fort Worth, Texas, April 24, 25, 26, 1906.

and hence I do not waste space in reproducing it, in as much as it is of no interest to my thousands of readers who are not members. A good program has been prepared and lots of social entertainment. But you bet I'll have a bully write-up of the meeting, as I have heretofore done for twenty years. So long.

DR. H. B. DECHARD, formerly of Galveston, has located in Dallas. Specialty, eye, ear, nose, and throat.

---

"Permit me to congratulate you on the excellence of the 'Red Back.' Fraternally yours," etc.—*Kent V. Kibbee, Editor Texas Courier Record-Medicine.*

---

DO NOT FAIL to read the arraignment of the A. M. A. management in two editorials from the *American Medical Journalist,* reproduced under "Abstracts." They hit the heart of the matter with a keen thrust. We have been gold-bricked, sure enough.

---

"O, DON'T DOCTOR!": DON'T fail to notify me when you change your address.

DON'T fail to notify me if you wish to discontinue your subscription, and don't fail to pay arrears.

DON'T fail to renew your subscription annually.

DON'T get mad when I send you a bill or draw on you.

DON'T fail to mention the "Red Back" in writing to advertisers.

---

"RED BACK" ADVERTISING PAID A HUNDRED FOLD TO ONE ADVERTISER.—Dr. J. W. Kenney's Sanitarium, San Antonio, now holds the second cover of the "Red Back," and attention is directed to the advertisement. Dr. Kenney writes: "My four dollar-and-a-half advertisement brought me more than *as many hundred* dollars during February. I also received many letters that will result in further business—one from Dr. ————, Pueblo, Mexico." The doctor is very successful with the "Lott Treatment" for morphinism and alcoholism, and stands A1 in the profession.

---

DR. E. B. BLAILOCK, Woodlawn, Texas, who represented Harrison county in the Legislature last session and who so ably and earnestly co-operated with and assisted the State Medical Association in its efforts for medical and sanitary legislation, is a candidate for Lieutenant-Governor. In his announcement he says he will champion the interest of the public health, which he regards as paramount to any other, and will aid the organized profession in their laudable efforts for sanitary laws. The readers of the "Red Back" should show their appreciation of his labors by voting for him.

The Mixed Board Bill, herewith presented, which was submitted to county societies for action before being presented to the House of Delegates at Fort Worth, was unanimously condemned by (this) Travis County Medical Society, and its delegate was instructed to oppose its adoption by the House.

And here I would remark, this important matter should not be disposed of by the House of Delegates—about 2 per cent of the membership—without giving the other 98 per cent a voice and a vote, as was the case with the adoption of a State Association journal in place of the Transactions.

---

"This Here Jones" (of the California Tentacle of the Octopus) harps continually on and derides and denounces the "individually owned, and conducted-for-profit-medical journals," meaning the independent medical press. I wish to be informed for what is the Octopus run, if not for profit? For amusement, or for Dr. Simmons' health? "This here Jones" says himself that its profit, annually (excess of income over expenses), is plus $40,000, and it is generally credited with an income of nearly a quarter-million dollars. Now, what is done with this $40,000 annual profit? It is invested in revenue-producing real estate and stocks, etc. For whose benefit? Has a dividend *ever* been declared?—not annually, as it should be, but—even once? One dividend would give each of the subscribers to the Octopus (said to be 20,000) a rebate of $2, which would reduce the subscription to $3 a year; and an annual dividend should enable the Association to furnish at least its transactions free to members, as we always did in Texas before the change was made to a journal without the knowledge or consent of the members. O, Jonesey, go chase yourself

## Figures and Tables

**Figure f1:**